# Comparison on the quality of sterile *Aedes aegypti* mosquitoes produced by either radiation-based sterile insect technique or *Wolbachia*-induced incompatible insect technique

**DOI:** 10.1371/journal.pone.0314683

**Published:** 2025-02-12

**Authors:** Pattamaporn Kittayapong, Suwannapa Ninphanomchai, Parinda Thayanukul, Jiraporn Yongyai, Wanitch Limohpasmanee

**Affiliations:** 1 Center of Excellence for Vectors and Vector-Borne Diseases, Faculty of Science, Mahidol University at Salaya, Nakhon Pathom, Thailand; 2 Department of Biology, Faculty of Science, Mahidol University, Bangkok, Thailand; 3 Thailand Institute of Nuclear Technology, Ministry of Higher Education, Science, Research and Innovation, Nakhon Nayok, Thailand; Instituto Leonidas e Maria Deane / Fundacao Oswaldo Cruz, BRAZIL

## Abstract

Novel and alternative vector control approaches using a sterile male-based release to suppress *Aedes aegypti* mosquito vectors have recently been tested in the field in many countries. These approaches included the sterile insect technique (SIT), incompatible insect technique (IIT), and a combination of both techniques. In this study, we conducted a series of experiments to compare the quality between radiation-based and *Wolbachia*-induced sterile males in terms of flight ability, sterility, mating competitiveness, survival rate, and longevity. *Aedes aegypti* mosquitoes irradiated at 50 Gy (SIT) and those trans-infected with *w*AlbB *Wolbachia* (IIT) were used for quality comparison. Our results showed that irradiated and *Wolbachia* trans-infected males were not significantly different in flight ability (*p* > 0.05) and both could induce sterility in wild-type females. In addition, although irradiation at 50 Gy or *Wolbachia* trans-infection reduced male mating competitiveness, combined irradiation and *Wolbachia w*AlbB trans-infection increased male competitiveness at the one-to-one ratio. Increasing the number of sterile males released could compensate for reduced competitiveness but it does not make them more competitive. Irradiation did not affect the survival and longevity of irradiated males, but it showed significant negative impacts on females (*p* < 0.05); while the opposite was observed in the case of *Wolbachia* infection, i.e., with significant increase in the survival rate of *Wolbachia* trans-infected males (*p* < 0.05), but both survival and longevity were reduced in *Wolbachia* trans-infected females with no significant impacts (*p* > 0.05). In conclusion, neither irradiation nor *Wolbachia* trans-infection significantly affected the quality of sterile males except their mating competitiveness; but this could compensate by increasing the number of sterile males released. Sterility could be induced by either 50 Gy irradiation or *w*AlbB trans-infection. Mating competitiveness results showed that a higher number of sterile males produced by irradiation need to be released in comparison to those produced by *Wolbachia* trans-infection. Our results should be useful for planning SIT, IIT, or a combination for *Ae*. *aegypti* vector control.

## Introduction

Mosquito-borne diseases are rapidly spreading to vast territories, putting at risk most of the world’s population. Major human arboviral pathogens, including dengue, chikungunya, yellow fever, and Zika, are transmitted by *Aedes aegypti* [1, 2]. Since there is an effective vaccine only for yellow fever [3], vector control initiatives in combination with educational approaches that engage the population to eliminate breeding sites, and the use of insecticides to suppress mosquito populations, are the only solutions available to fight epidemic outbreaks [4]. However, since vector control strategies that rely on insecticides were unsustainable over the long term, due to surges of resistant vector populations [5], several genetic control methods, including the *Wolbachia* technology, have been suggested as potential tools for the population control of *Ae*. *aegypti*, and some of them are currently being tested in the field [6].

Successful implementation of the Sterile Insect Technique (SIT), as part of an Integrated Pest Management (IPM) approach, for the control of several insect pests of the agricultural industry has encouraged significant efforts to develop analogous techniques for mosquito control [7, 8]. Two environmentally friendly control strategies, namely the radiation-based Sterile Insect Technique (SIT) and the *Wolbachia*-induced Incompatible Insect Technique (IIT) are currently being developed in several laboratories worldwide [9]. Both SIT and IIT are based on a male release that aims to introduce sterility or lethality in the target population [10]. SIT relies on mass-rearing production, sterilization, and the recurrent release of sterile males of the targeted species that are typically attained by radiation in a way that does not impair male mating and insemination capabilities. Radiation caused germ-cell chromosome fragmentation, leading to dominant lethal mutations, which then resulting in imbalanced gametes, inhibition of mitosis and ultimate death of the embryo [11]. Hence, when wild females mate with sterile males, the eggs do not hatch [10] resulting in population suppression over the generations. Irradiation-induced dominant lethal mutations in the germ cells had sterilization effect on fertilized embryos, and was the main cause of male sterilization in the SIT approach [10]. The advantages of SIT over other pest control approaches are that this technique is species-specific and environmentally friendly [12], and resistance is less likely to evolve [13]. SIT has been applied successfully for several non-tephritid insect pests, including the New World screw worm *Cochliomyia hominivorax* (Coquerel), several species of tsetse fly (*Glossina* spp.), the codling moth *Cydia pomonella* (L.) [14–16], and mosquitoes [7, 17].

An alternative is IIT, a method involves the release of *Wolbachia*-harboring insects in a natural habitat, where they can mate with wild females [18]. IIT also relies on the principle of reducing female fertility by utilizing endosymbiotic bacteria from the genus *Wolbachia*, instead of radiation, to induce a form of reproductive incompatibility known as cytoplasmic incompatibility (CI) in wild females [19]. *Wolbachia* induced a form of embryonic death, so called CI [20], resulting from sperm-egg incompatibility which occurred when *Wolbachia*-infected males mated with uninfected females or females infected with an incompatible *Wolbachia* strain. Cytoplasmic Incompatibility was thus exploited as a source of sterility through the IIT strategy [21]. In general, the *Wolbachia*-based approach aims at either replacing a target population with a strain having reduced vector competence (population replacement) [22], or suppressing mosquito population below the threshold required for disease transmission (population suppression), the latter being similar to SIT in its application and effects. IIT field trials have resulted in the historical success of eradicating *Culex pipiens* [23] and the recent success in effectively suppressing *Aedes aegypti* and *Aedes albopictus* [24, 25].

In the absence of an efficient and robust method of sex separation for *Aedes* species, although pupa size is about as efficient and robust as natural means can be but it is not perfect. Therefore, it was recently suggested that irradiation (SIT) and *Wolbachia* (IIT) can be combined, which may eliminate the risks associated with the presence of a few females in sterile male batches being released in the field to suppress a target population [26]. The effectiveness of any male release-based approach for population suppression of a mosquito vector depends on the availability of appropriate numbers of sterile males, and any strain which is a candidate for sterile male releases should undergo quality analysis and be tested with respect to its productivity and the quality of the males, particularly regarding their mating competitiveness which is well defined for fruit flies [27]. So far, however, there is a lack of comparative assessment of these strategies under the same controlled conditions [28].

**Fig 1:**
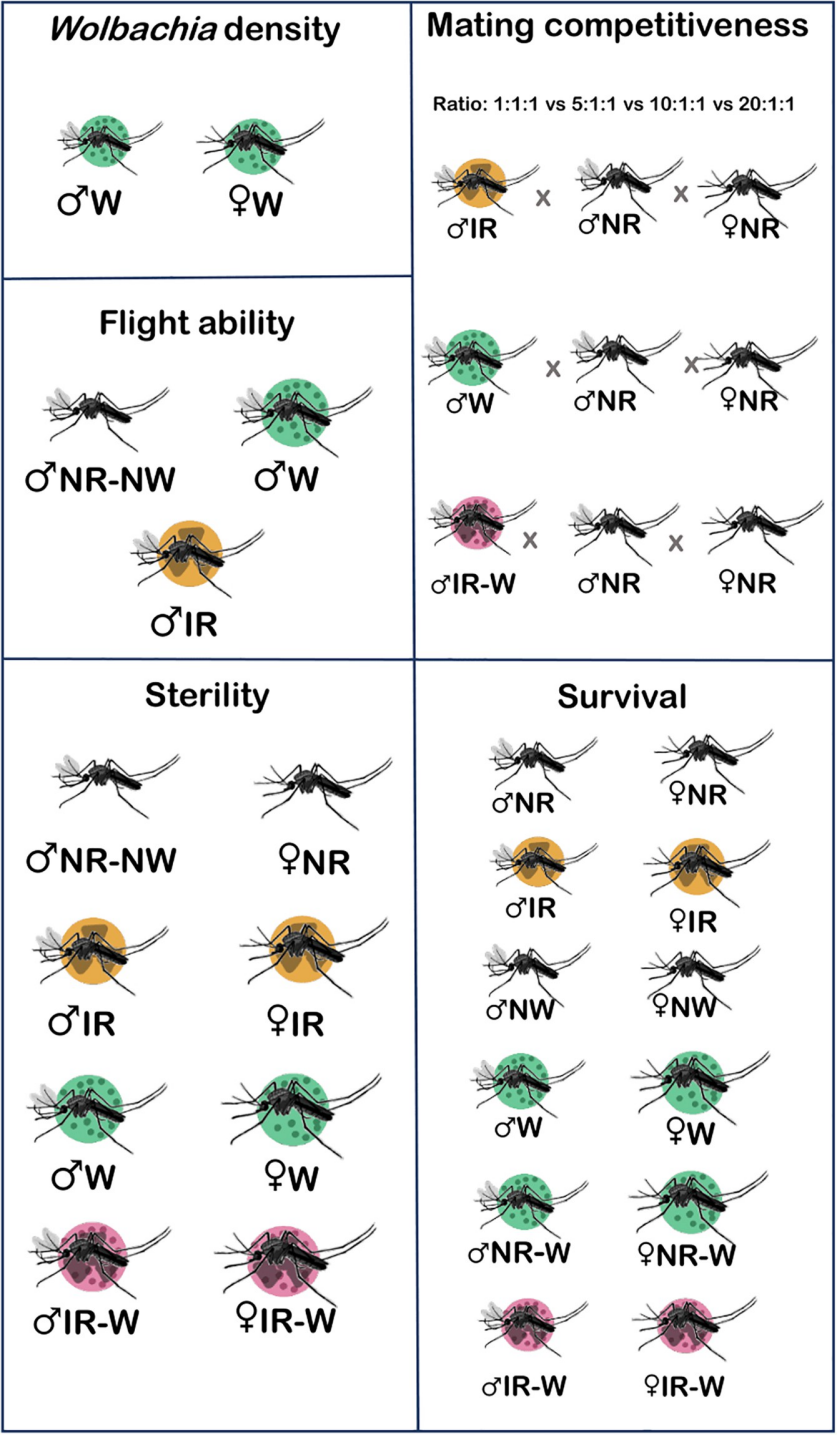
Overall experiments conducted with non-irradiated *Wolbachia* uninfected (nr-nw), irradiated (r) *and Wolbachia* trans-infected (w) *Aedes aegypti* males and females.

In this study, we assessed and compared the quality of sterile *Ae*. *aegypti* males produced by either SIT or IIT or combined SIT/IIT by examining the *Wolbachia* density, flight ability, sterility, mating competitiveness, survival rate, and longevity under laboratory-controlled conditions (Fig 1). Results from this study should provide important insights on the relative effectiveness of SIT and IIT for the control of natural populations of *Ae*. *aegypti* mosquito vectors.

## Materials and methods

### Mosquito colony and rearing methods

The *Aedes aegypti* mosquitoes used in the present study were originally collected from several communities in Chatuchak District, Bangkok. A *Wolbachia* trans-infected *Ae*. *aegypti* colony was obtained from direct microinjection using this colony and the *w*AlbB *Wolbachia* strain from the *Ae*. *albopictus* colony originating from Plaeng Yao District, Chachoengsao Province, using the method described by Ruang-areerate and Kittayapong [29]. The establishment and characteristics of the *Wolbachia* trans-infected mosquitoes have been demonstrated [30, 31].

In these experiments, mosquitoes were reared in an aluminum mass-rearing cage sized 40 cm x 100 cm x 150 cm. Mosquitoes were reared in a screened climatic control insectary at the Center of Excellence for Vectors and Vector-Borne Diseases, Faculty of Science, Mahidol University at Salaya, Nakhon Pathom, Thailand, with 75 ± 2% relative humidity, 27 ± 2°C, and a photoperiod of L12:D12, and were fed with 10% sucrose solution.

For egg-laying, female mosquitoes were fed with pig blood using the Hemotek membrane feeding system (Hemotek Ltd., UK) for 3–4 consecutive days after mating. The blood, obtained from a qualified slaughterhouse, was treated with 15 mM EDTA (SCHARLAU, Spain). Egg papers were placed in the containers inside the cage 1–3 days following blood-feeding. After 3–4 days, the egg papers were then collected, dried for 1–3 days at room temperature, and transferred into glass containers filled with deionized water without any nutrients provided for egg hatching with screw-top covers. After the eggs were hatched into the first-instar larvae, they were counted and transferred into plastic trays sized 30 cm x 40 cm x 5 cm, each containing about 2,000 larvae. After egg hatching, a larval diet was provided daily, at a total quantity of 6.5 g as described by Kittayapong et al. [31]. No larval diet was given when larvae reached the pupal stage, which took about 6–7 days. A dropper was used to remove larvae that were mixed with pupae, and pupae were then placed in plastic containers prior to sex separation.

### Sex separation and irradiation procedures

*Aedes aegypti* pupae were sourced by using the local pupal sex separator modified from the larval-pupal sex separator [32]. Pupae aged from 24–48 hours were placed in plastic containers, each being 122.66 cm^3^ in volume (diameter 12.5 cm, height 14.5 cm) and with 62 ml of water, prior to transportation to the radiation source. Five hundred pupae were placed in water inside each container. The containers were covered with lid that was attached with mesh in order to allow air flow into the container. Male and female pupae were placed in separated containers. These plastic containers were placed in a styroform box prior to transportation by air-conditioned car from the laboratory at Mahidol University Salaya Campus, Nakhon Pathom Province to the Thailand Institute of Nuclear Technology (Public Organization) (TINT), Nakhon Nayok Province, which is located 112 km away or about 4 hours round-trip. The containers with 24–48 hour pupae submerged under water were placed inside an irradiator. Using a Colbalt-60 (Gammar Chamber 5000, Board of Radiation and Isotope Technology (BRIT), DAE, Mumbai, India), an irradiation dose of 50 Gy which required 45 seconds at the rate of 66.66 Gy per minute (residual fertility of 88.19 ± 10.87%), was applied by qualified staff at TINT.

After irradiation, the irradiated male and female pupae were transported back to the laboratory for further experiments. The irradiated males were used for *Wolbachia* density, flight ability, sterility, mating competitiveness, and survival and longevity while the irradiated females were used for experiments on *Wolbachia* density, sterility, survival, and longevity. Then small plastic containers holding irradiated pupae were put in aluminum cages prior to adult emergence, and a 10% sucrose solution was provided. Irradiated male and female pupae were placed in separated cages.

### Method for detection of *Wolbachia*

Five to ten *Wolbachia* trans-infected *Ae*. *aegypti* females were taken from mosquito cages to analyze for *Wolbachia* after egg-laying using PCR [33]. Each generation, mosquitoes were ground in 100 μl STE (100 mM NaCl, 10 mM Tris-HCl, 1 mM EDTA, pH 8.0) at 3,000 rpm for 3–5 min using Tissue Lyser II, with the aid of a 3 mm bead. The grinding solution was heated at 95°C for 10 min and then centrifuged at 14,000 rpm for 1 min. *Wolbachia* DNA was amplified in a 20 μl solution of 2 μl of MgCl_2_, 1 μl of 50mM MgCl_2_, 1 μl of 10mM dNTP, 0.5 μl of forward and reverse primers, 1 μl of Taq DNA polymerase (Invitrogen, USA), and 12 μl of distilled water. PCR was carried out at 95°C for 3 min. followed by 35 cycles of 95°C for 1 min, 50°C for 1 min, and 72°C for 1 min. PCR products were electrophoresed on 2% agarose gel in TAE buffer strained with ethidium bromide. DNA bands were visualized under UV light inside a GelDoc machine. The qPCR analysis was performed using the relative quantification of the *wsp* gene (*Wolbachia* surface protein) of *Wolbachia* [34]. General primers used to detect *Wolbachia* were *wsp* 81F (5’-TGG TCC AAT AAG TGA AGA AAC- 3’) and *wsp* 691R (5’- AAA AAT TAA ACG CTA CTC CA-3’). Mosquitoes that tested positive for *Wolbachia* were further tested by PCR using specific primers for *w*AlbB: *wsp* 81F (5’-TGG TCC AAT AAG TGA AGA AAC-30) and *wsp* 522R (5’-ACC AGC TTT TGC TTG ATA-30). The specific PCR screening for *w*AlbB was carried out in order to confirm that *Wolbachia* trans-infection was successful and *w*AlbB could be established in our mosquito colony.

### Method for detection of *Wolbachia* density

In these experiments, a comparison was done between 1) irradiated *Wolbachia* trans-infected *Ae*. *aegypti* (♂ ir-w) males and control (♂ nr-w) males, and 2) irradiated *Wolbachia* trans-infected *Ae*. *aegypti* (♀ ir-w) females and control (♀ nr-w) females. *Wolbachia* density was detected with 14 days old *w*AlbB *Ae*. *aegypti* males and females irradiated at 50 Gy. Genomic DNA was extracted from a whole mosquito body using DNeasy Blood & Tissue Kit (Qiagen, Germany). Each sample was measured by a quantitative real-time PCR (qPCR) with two replications. The homothorax gene (HTH) was used as a reference gene of mosquito [35]. The cycle threshold (Ct) of *Wolbachia* surface protein and homothorax gene was recorded in each sample. *Wolbachia* density was calculated using the following equation [36].


$$2^{\Delta \text{Ct}}={\text{C}}_{\text{t}Ae.aegypti}-{\text{C}}_{\text{t}Wolbachia}$$

The qPCR mixture was prepared by using iTaq Universal SYBR Green Supermix (BIO-RAD, USA) according to the manufacturer’s recommendation, and a qPCR was performed using the following program: 45°C for 10 min, initial denaturation at 95°C for 3 min, followed by 40 cycles of 95°C for 5 sec, 59°C for 30 sec and 74.5°C for 10 sec.

### Flight ability test of SIT or IIT mosquitoes

The flight ability test was done as described by Culbert [37] with modification of the cotton moistened with a 10% sucrose solution, placed on top of the device as a lure instead of one small pellet of BG lure (Biogents, Regensburg, Germany). A mouth aspirator was used to aspirate either 100 irradiated (SIT) or *Wolbachia* trans-infected (IIT) *Ae*. *aegypti* males into the flight test devices via a small 1 cm hole at the bottom of the device. A 12 V fan was connected to the battery and then switched on to start the experiment. After two hours, the fan was turned off and the experiment was terminated. The device was then refrigerated at -20°C for 5–10 minutes in order to immobilize the mosquitoes. The number of mosquitoes that successfully escaped the flight tubes and the number of those that still remained within the tubes were counted. The escape rate was calculated by dividing the number of escaped males by the total number of males. Comparison of the escape rate was done between irradiated and non-irradiated (control), and *Wolbachia* trans-infected *Ae*. *aegypti* and non-*Wolbachia* trans-infected (control) males.

### Sterility test of SIT or IIT mosquitoes

**Fig 2:**
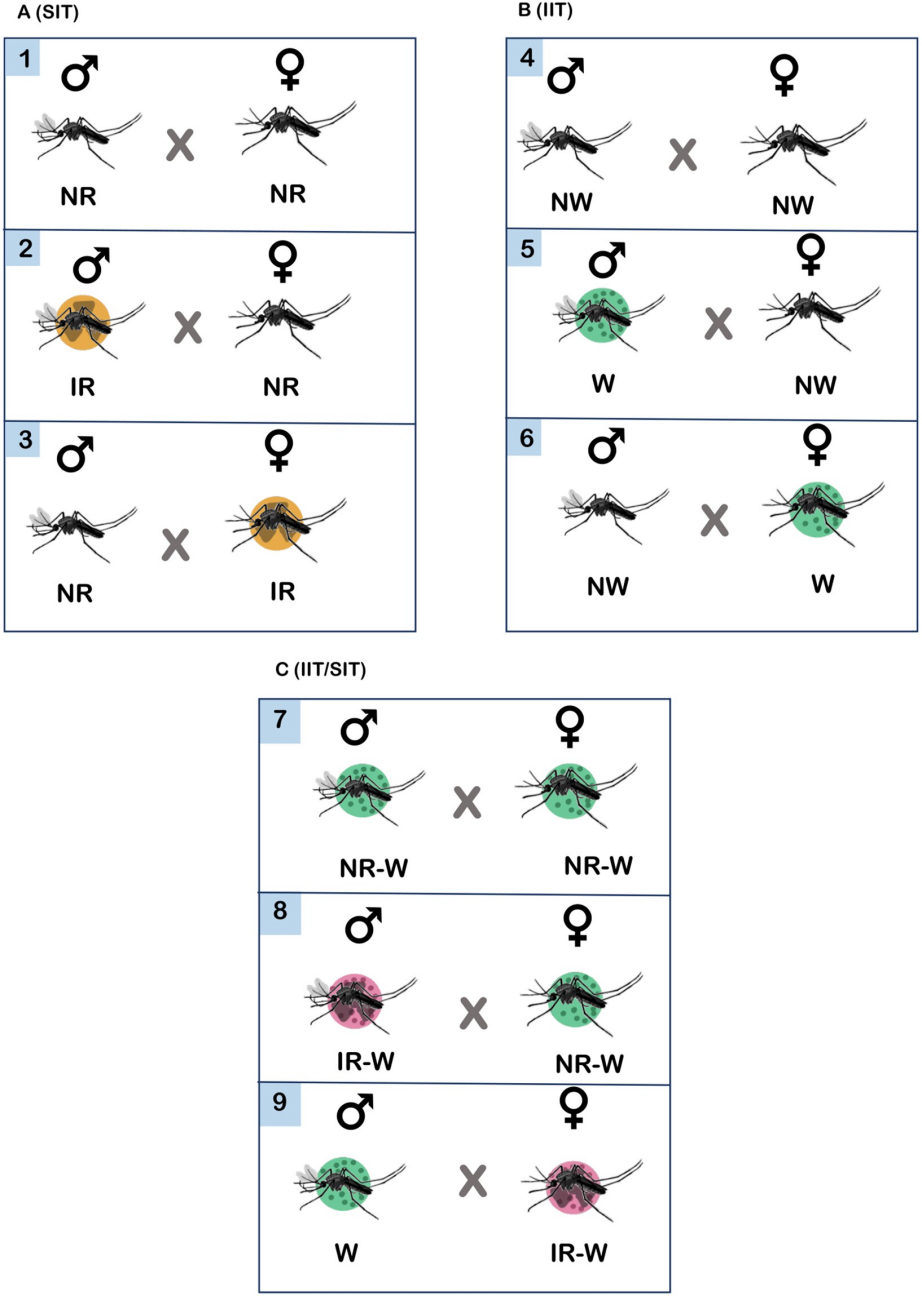
Experiment on sterility of cross-mating pairs between non-irradiated (nr), and irradiated (ir) *Aedes aegypti* mosquitoes (A), *Wolbachia* uninfected (nw) and *Wolbachia* trans-infected (w) *Aedes aegypti* mosquitoes (B), and non-irradiated *Wolbachia* trans-infected (nr-w) and irradiated *Wolbachia* trans-infected (ir-w) *Aedes aegypti* mosquitoes (C).

Preliminary experiments were set up to test the sterility of 50 Gy irradiated (SIT) *Ae*. *aegypti* male and female mosquitoes (S1 Fig, Supplementary data). In the experiments, the cross-mating pair between the irradiated males (♂ ir) and the non-irradiated females (♀ nr), and the non-irradiated males (♂ nr) and irradiated females (♀ ir), in a ratio of 1:1 were conducted in 30 cm × 30 cm × 30 cm cages, with a 10% sucrose solution provided (Fig 2A). The non-irradiated males (♂ nr) and females (♀ nr) with the same ratio were also introduced into the cage and used for control. The same experiments were conducted with *Wolbachia* trans-infected (IIT) *Ae*. *aegypti* males (♂ w) and females (♀ w) (Fig 2B) in which *Wolbachia*-uninfected males (♂ nw) and females (♀ nw) were introduced into the cage and used for control. Lastly, the same experiments were also conducted with irradiated *Wolbachia* trans-infected (IIT/SIT) *Ae*. *aegypti* males (♂ ir-w) and females (♀ ir-w) (Fig 2C), in which non-irradiated *Wolbachia* trans-infected males (♂ nr-w) and females (♀ nr-w) were used for control. Symbols of mosquitoes used in this article, and its descriptions were summarized in Table 1.

**Table 1:** Summary of symbols and description of *Aedes aegypti* mosquitoes used in this study.

Symbols	Description
♂ nw	*Wolbachia*-uninfected *Aedes aegypti* males
♀ nw	*Wolbachia*-uninfected *Aedes aegypti* females
♂ w	*Wolbachia* trans-infected *Aedes aegypti* males
♀ w	*Wolbachia* trans-infected *Aedes aegypti* females
♂ nr-nw	Non-irradiated *Wolbachia*-uninfected *Aedes aegypti* males
♀ nr-nw	Non-irradiated *Wolbachia*-uninfected *Aedes aegypti* females
♂ nr-w	Non-irradiated *Wolbachia* trans-infected *Aedes aegypti* males
♀ nr-w	Non-irradiated *Wolbachia* trans-infected *Aedes aegypti* females
♂ ir-w	Irradiated *Wolbachia* trans-infected *Aedes aegypti* males
♀ ir-w	Irradiated *Wolbachia* trans-infected *Aedes aegypti* females
♂ nr	Non-irradiated wild type *Aedes aegypti* males
♀ nr	Non-irradiated wild type *Aedes aegypti* females
♂ ir	Irradiated *Wolbachia*-uninfected *Aedes aegypti* males
♀ ir	Irradiated *Wolbachia*-uninfected *Aedes aegypti* females

The mosquitoes were freely mated in the cages for 2–3 days. The females were then blood-fed using the Hemotek blood-feeding system (Hemotek Ltd., UK). Each blood-feeding period lasted 1–2 hours, and the Hemotek blood-feeding unit with new blood was re-introduced within 2–3 consecutive days. Blood-fed female mosquitoes were individually separated and placed in a plastic tube 7 cm^3^ in volume (diameter 3 cm, height 5.5 cm). Insemination of blood-engorged females were studied by presence or absence of sperm in spermathecae by phase contrast microscopy through the distended abdomen, after the intersegmental membranes were stretched by ventral placement of a glass cover slip [38]. Egg paper was placed over wet cotton inside each plastic tube for oviposition. After 3–4 days, the egg paper from each female mosquito was collected and the eggs were counted. Then the egg paper was dried and transferred into a glass container containing deionized water for hatching, as previously described. The number of hatched and un-hatched eggs from each individual female mosquito was recorded. The un-hatched eggs represented the sterility of the tested mosquitoes.

### Mating competitiveness of SIT or IIT mosquitoes

**Fig 3:**
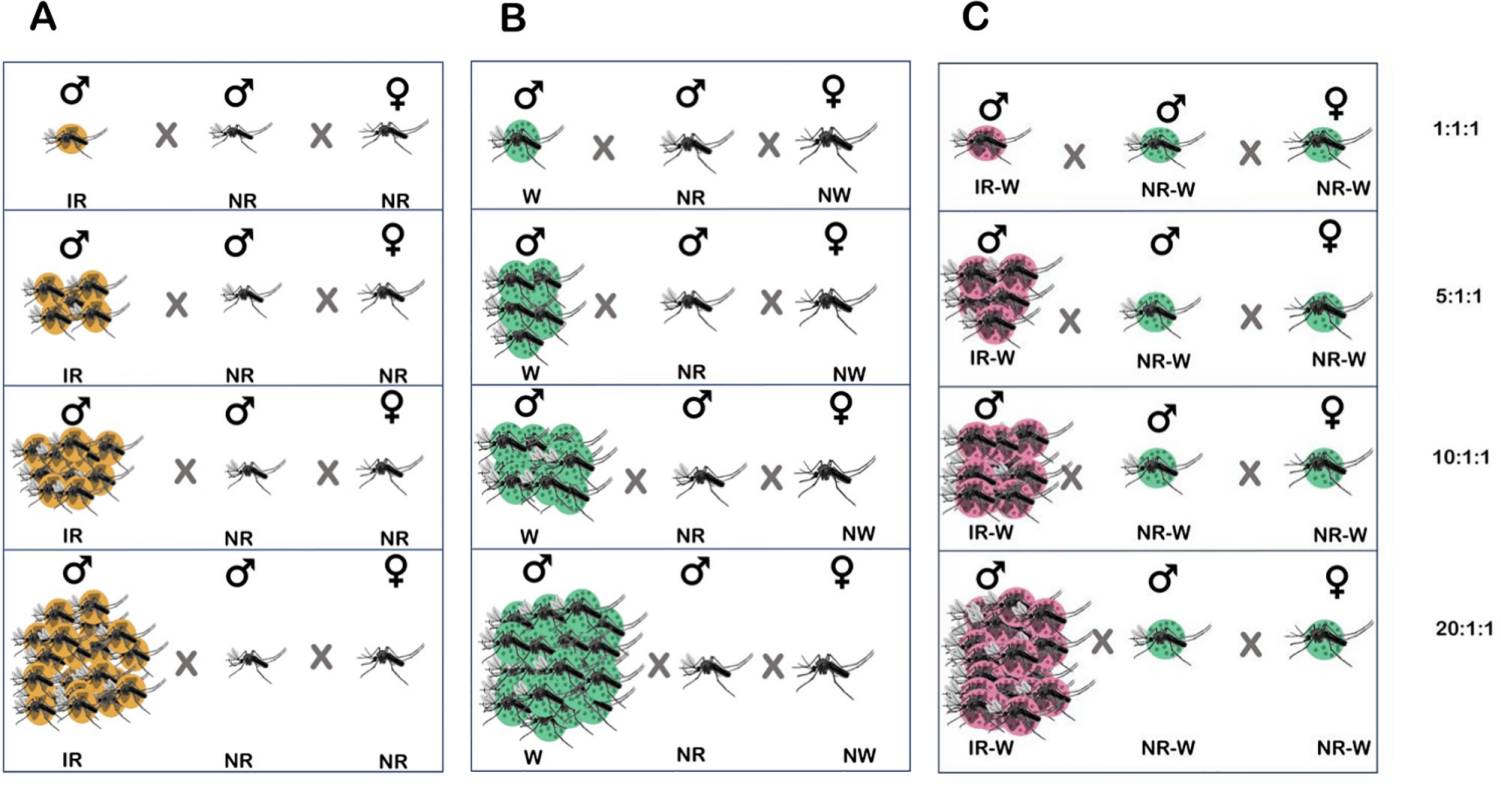
Experiment on competiveness between irradiated males (♂ ir) vs non-irradiated wild type males (♂ nr), *Wolbachia* trans-infected males (♂ w) vs *Wolbachia*-uninfected wild type males (♂ nw), and irradiated *Wolbachia* trans-infected males (♂ ir-w) vs non-irradiated wild type males (♂ nr-w), at the ratios of 1:1:1, 5:1:1, 10:1:1 and 20:1:1.

An experiment was conducted to determine the mating competitiveness of irradiated (SIT) *Ae*. *aegypti* males after being irradiated at 50 Gy. The mating ratios between ♂ ir vs ♂ nr and ♀ nr were 1:1:1, 5:1:1, 10:1:1, and 20:1:1 respectively. Each non-irradiated wild type female was put in a separate 30 cm x 30 cm x 30 cm cage, and one non-irradiated wild type male and 1, 5, 10, or 20 irradiated males were introduced into each cage respectively [30]. In these experiments, mating of ♂ ir vs ♂ nr and ♀ nr were investigated at the ♂ ir: ♂ nr: ♀ nr ratios of 1:1:1, 5:1:1, 10:1:1, 20:1:1 (Fig 3A). Then ♀ nr were blood-fed, and they were individually separated into a small plastic cup with oviposition paper provided inside the cup. The number of eggs laid per female and the number of hatched eggs were counted, and the egg hatch rate was evaluated for each mating ratio. These mosquito cages were left in an insectary at 75 ± 2% humidity, 27 ± 2°C temperature, and a photoperiod of L12:D12. A 10% sucrose solution was provided as food source for adult mosquitoes, and pig blood was provided as blood meals for females about 3–4 days after male introduction. Oviposition cups were introduced to each mosquito cage to collect eggs. After 3–4 days, each egg paper in the oviposition cups was collected, dried at room temperature, and then the eggs were counted before hatching in deionized water. The total number of eggs hatched and the egg hatch rate of each egg batch were recorded to determine the male mating competitiveness. The same experiments were conducted to determine the mating competitiveness of *Wolbachia* trans-infected (IIT) *Ae*. *aegypti* males. The mating ratios between ♂w vs ♂nr-w and ♀nr-w were 1:1:1, 5:1:1, 10:1:1, and 20:1:1 respectively (Fig 3B). Lastly, the same experiments were conducted to determine the mating competitiveness of irradiated *Wolbachia* trans-infected (IIT/SIT) *Ae*. *aegypti* males. The mating ratios between ♂ir-w vs ♂nr-w and ♀nr-w were 1:1:1, 5:1:1, 10:1:1, and 20:1:1 respectively (Fig 3C). Thirty replicates were conducted for each experiment.

Thirty replicates of the male mating competitiveness index (C) was calculated [39] and the induced sterility (IS) was assessed in order to evaluate the effect of sterile male releases [39, 40].

### Survival rate and longevity of SIT or IIT mosquitoes

The emerged irradiated (SIT) males and females; ♂ ir, ♀ ir, *Wolbachia* trans-infected (IIT) males and females; ♂ w, ♀ w, and irradiated *Wolbachia* trans-infected males and females; ♂ ir-w, ♀ ir-w mosquitoes, were separately introduced into a 30 cm x 30 cm x 30 cm cage, with 10% sucrose solution provided. Each cage contained 30 mosquitoes per cage. The cages were placed in an insectary at a temperature of 27 ± 2°C, 75 ± 2% humidity, and a photoperiod of L12:D12. The number of dead male and female mosquitoes was daily observed and recorded. The dead mosquitoes were then removed from the cage.

### Statistical analysis

Data was entered and cleaned using Microsoft Office Excel 2016 and statistical analysis was performed further using SPSS 18.0 (Mahidol University License (Chicago, SPSS Inc.). *Wolbachia* density and flight ability, were analyzed by using ANOVA and paired-sample t test. Induce sterility (IS) and Fried Index (C) were analyzed by using one sample t-test. Survival was analyzed by using Kaplan Meir survival. P-values of less or equal to 0.05 were considered significant.

## Results

### Comparison of *Wolbachia* density between irradiated (SIT) and *Wolbachia* trans-infected (IIT) *Aedes aegypti* males and females

In this study, detection of the *w*AlbB strain was 100% in *Wolbachia* trans-infected male and female mosquitoes (S1 Table, Supplementary data). Our results showed that *Wolbachia* density of ♂ ir-w was significantly reduced when compared to those of control males (♂ nr-w) (*p* < 0.05) (S2 Table, Supplementary data). For ♀ ir-w, *Wolbachia* density was reduced when compared to those of control females (♀ nr-w), but this difference was not statistically significant (*p* > 0.05) (S2 Table, Supplementary data).

### Comparison of flight ability between irradiated (SIT) and *Wolbachia* trans-infected (IIT) *Aedes aegypti* males

In these experiments, a comparison was done between 1) ♂ ir vs ♂ nr-nw and 2) ♂ w vs ♂ nr-nw by using a Turkey HSD post-hoc test. Results from three replicates of each experiment showed that the escape rate of ♂ ir and ♂ w were not different than those of the control (*p* > 0.05) (Table 2). With the previous results, when the escape rate of ♂ ir was compared to those of ♂ w, it was found that there was no difference between these two males (*p* > 0.05) (Table 2).

**Table 2:** Turkey HSD post-hoc test of the escape rate between irradiated *Aedes aegypti* (♂ ir) males and *Wolbachia* trans-infected *Aedes aegypti* (♂ w) males when compared to those of the control (♂ nr-nw) males.

Dependent variable	Experiments	Experiments	Mean difference	SE	Sig	95% CI
Escape rate	♂ nr-nw	♂ ir	0.03000	0.1414	0.165	-0.01–0.07
	♂ nr-nw	♂ w	0.00333	0.1414	0.970	-0.04–0.05
	♂ ir	♂ nr-nw	-0.03000	0.1414	0.165	-0.07–0.01
	♂ ir	♂ w	-0.02667	0.1414	0.223	-0.07–0.02
	♂ w	♂ nr-nw	-0.00333	0.1414	0.970	-0.04–0.04
	♂ w	♂ ir	0.02667	0.1414	0.223	-0.02–0.07

* Significant difference at *p* < 0.05

In conclusion, the flight ability of ♂ ir and ♂ w of *Ae*. *aegypti* was not significantly different from those of the control; therefore, irradiation dose of 50 Gy and *Wolbachia* trans-infection had no impact on the flight ability.

### Comparison of sterility between irradiated (SIT) and *Wolbachia* trans-infected (IIT) *Aedes aegypti* mosquitoes

Cross-mating between ♂ ir x ♀ nr, ♂ nr x ♀ ir, ♂ w x ♀ nw, and ♂ nw x ♀ w were investigated. For the last crosses between ♂ ir-w x ♀ nr-w and ♂ nr-w x ♀ ir-w, the data were obtained from Kittayapong et al. [31] for comparison. The number of total eggs and number of hatched eggs were recorded from each cross-mating pair; then the egg hatch rate was calculated and compared.

Results showed that for irradiated (SIT) mosquitoes, both ♂ ir and ♀ ir were completely sterile since no eggs were hatched when they were cross-mating with ♀ nr and ♂ nr respectively (Table 3). For *Wolbachia* trans-infected mosquitoes, it was found that ♂ w were completely sterile when they were cross-mated with ♀ nw as no eggs were hatched. However, it was not the case for ♀ w since they could still lay eggs and the eggs could hatch (Table 3). For irradiated *Wolbachia* trans-infected mosquitoes, results showed that ♂ ir-w was not completely sterile as it still induced a very low egg hatch rate when they were cross-mating with ♀ nr-w (Table 3). In contrary, ♀ ir-w was completely sterile and they could lay no eggs when they were cross-mating with ♂ nr-w.

**Table 3:** Analysis of variance of total number of eggs, hatched eggs, and egg hatch rate 1) between ♂ ir x ♀ nr vs ♂ nr x ♀ ir, 2) between ♂ w x ♀ nw vs ♂ nw x ♀ w, and 3) between ♂ ir-w x ♀ nr-w vs ♂ nr-w x ♀ ir-w.

Mating pair	No. Rep.	N	Mean ± SD	95% CI	T	df	*p*
**Total eggs**							
♂ ir x ♀ nr	60	355	88.75 ± 24.13	50.35–127.15	7.356	3	0.005*
♂ nr x ♀ ir	60	0	0.00 ± 0.00				
♂ w x ♀ nw	60	4,579	76.32 ± 29.65	-5.22–14.42	0.937	59	0.353
♂ nw x ♀ w	60	4,303	71.72 ± 26.24				
♂ ir-w x ♀ nr-w^(a)^	24	1,110	1,341.13 ± 431.61	1,158.87–1,523.38	15.22	23	0.001*
♂ nr-w x ♀ ir-w^(b)^	24	1,105	0.00 ± 0.00				
**Hatched eggs**							
♂ ir x ♀ nr	60	355	0.00 ± 0.00	NA	NA	NA	NA
♂ nr x ♀ ir	60	0	0.00 ± 0.00				
♂ w x ♀ nw	60	4,579	0.00 ± 0.00	-58.92–-40.52	-10.812	59	0.000*
♂ nw x ♀ w	60	4,303	49.72 ± 35.62				
♂ ir-w x ♀ nr-w^(a)^	24	1,110	1.04 ± 2.18	0.12–1.96	2.35	23	0.028*
♂ nr-w x ♀ ir-w^(b)^	24	1,105	0.00 ± 0.00				
**Egg hatch rate**							
♂ ir x ♀ nr	60	355	0.00 ± 0.00	NA	NA	NA	NA
♂ nr x ♀ ir	60	0	0.00 ± 0.00				
♂ w x ♀ nw	60	4,579	0.00 ± 0.00	-0.75–-0.54	-12.449	59	0.000*
♂ nw x ♀ w	60	4,303	0.65 ± 0.40				
♂ ir-w x ♀ nr-w^(a)^	24	1,110	0.07 ± 0.13	0.01–0.12	2.60	23	0.016*
♂ nr-w x ♀ ir-w^(b)^	24	1,105	0.00 ± 0.00				

* Significant difference at *p* < 0.05,

^**(a), (b)**^ Data from Kittayapong et al. (2018) for comparison

In conclusion, complete sterility (no hatched eggs) could be induced by either irradiation or *Wolbachia*-infection in *Ae*. *aegypti* males. However, complete sterility in females was induced by either irradiation alone or by a combination of irradiation and *Wolbachia* infection. When irradiation and *Wolbachia* infection were applied at the same time, complete sterility was obviously detected in females and nearly complete sterility was observed in males.

### Comparison of mating competitiveness between irradiated (SIT) and *Wolbachia* trans-infected (IIT) *Aedes aegypti* males

From experiments conducted with ♂ ir, with mating of ♂ ir x ♂ nr x ♀ nr at the ♂ ir: ♂ nr: ♀ nr ratios of 1:1:1, 5:1:1, 10:1:1, 20:1:1, results showed that the highest mean egg hatch rate was observed at the ratio of 1:1:1, followed by the ratio of 5:1:1, the ratio of 10:1:1, and the ratio of 20:1:1 respectively (Table 4). When Induced Sterility (IS) from each mating ratio was compared by using one sample t-test, with the theory value when ♀ ir were completely sterile (IS = 100), it was found that IS was significantly increased when the mating ratio increased (Table 4). When Fried Index (C) from each mating ratio was compared by using one sample t-test, with the theory value when the irradiated males were as competitive as the wild non-irradiated males (C = 1), it was found that the C value was increased when the mating ratio increased (Table 4). It was noticed that the C value was equal to or higher than 1 when the mating ratio was from 10:1:1 onwards.

**Table 4:** Competiveness between irradiated males (♂ ir) vs non-irradiated wild type males (♂ nr), *Wolbachia* trans-infected males (♂ w) vs *Wolbachia*-uninfected wild type males (♂ nw), and irradiated *Wolbachia* trans-infected males (♂ ir-w) vs non-irradiated wild type males (♂ nr-w), at the ratios of 1:1:1, 5:1:1, 10:1:1 and 20:1:1.

Ratio	No. Rep.	Total eggs (Mean ± SD)	Total hatched eggs (Mean ± SD)	Egg hatch rate (Mean ± SD)	Induced sterility (IS) (Mean ± 95%CI)	Fried Index (C) (Mean ± 95%CI)
**♂ ir:♂ nr:♀ nr**						
1:1:1	30	2,494 (83.13 ± 14.38)	1,972 (65.73 ± 12.68)	0.80 (0.80 ± 0.10)	99.12 (-0.92–-0.84)*	0.17 (-0.90–-0.76)*
5:1:1	30	2,464 (82.07 ± 26.58)	834 (27.80 ± 22.27)	0.33 (0.33 ± 0.20)	99.64 (-0.45–-0.28)*	0.80 (-0.47–0.08)
10:1:1	30	2,350 (78.33 ± 26.11)	299 (9.97 ± 6.87)	0.13 (0.13 ± 0.08)	99.85 (-0.18–-0.11)*	1.00 (-0.22–0.23)
20:1:1	30	2,157 (71.90 ± 26.68)	75 (2.50 ± 2.00)	0.03 (0.03 ± 0.02)	99.96 (-0.05–-0.03)*	1.79 (0.63–0.95)*
**♂ w:♂ nw:♀nw**						
1:1:1	30	2,103 (70.10 ± 15.86)	1,342 (44.73 ± 14.62)	0.64 (0.64 ± 0.16)	99.20 (-0.87–-0.72)*	0.35 (-0.79–-0.51)
5:1:1	30	2,026 (67.53 ± 25.67)	589 (19.63 ± 14.43)	0.30 (0.30 ± 0.17)	99.63 (-0.45–-0.29)*	0.71 (-0.58–-0.00)*
10:1:1	30	1,537 (51.23 ± 29.01)	135 (4.50 ± 2.90)	0.09 (0.09 ± 0.03)	99.89 (-0.12–-0.10)*	1.21 (0.05–0.37)*
20:1:1	30	1,589 (52.97± 22.70)	65 (2.17 ± 1.12)	0.04 (0.04 ± 0.01)	99.95 (-0.05–-0.04)*	2.05 (0.78–1.32)*
**♂ ir-w:♂ nr-w:♀ nr-w** ^(a)^						
1:1:1	30	1,069 (35.63 ± 9.87)	640 (21.33 ± 9.03)	0.60 (0.59 ± 0.17)	99.34 (-0.73–-0.59)*	0.71 (-0.56–-0.03)*
5:1:1	30	1,308 (43.60 ± 1.17)	235 (7.83 ± 2.74)	0.18 (0.18 ± 0.05)	99.80 (-0.22–-0.18)*	0.86 (-0.25–-0.02)*
10:1:1	30	1,638 (54.60 ± 8.78)	237 (7.90 ± 3.98)	0.14 (0.15 ± 0.08)	99.83 (-0.20–-0.13)*	0.70 (-0.46–-0.15)*
20:1:1	30	1,396 (46.53 ± 17.17)	52 (1.73 ± 0.91)	0.04 (0.04 ±0.03)	99.95 (-0.06–-0.04)*	1.37 (0.10–0.64)*

* Significant difference at *p* < 0.05,

^**(a)**^ Data from Kittayapong et al. (2019) for comparison

The same experiments were conducted with ♂ w, with mating of ♂ w x ♂ nw x ♀ nw at the ♂ w: ♂ nw: ♀ nw ratios of 1:1:1, 5:1:1, 10:1:1, 20:1:1. Results showed that the highest egg hatch rate was found at the ratio of 1:1:1, followed by the ratio of 5:1:1 (Table 4). When Induced Sterility (IS) from each mating ratio was compared, it was found that IS was significantly increased when the mating ratio increased (Table 4). When Fried Index (C) from each mating ratio was compared, it was found that C significantly increased and its value was higher than 1 when the mating ratio was from 10:1:1 onwards (Table 4).

The same experiments were conducted with ♂ ir-w, with mating of ♂ ir-w x ♂ nr-w x ♀ nr-w at the ♂ ir-w: ♂ nr-w: ♀ nr-w ratios of 1:1:1, 5:1:1, 10:1:1, 20:1:1. Results showed that the highest egg hatch rate was found at the ratio of 1:1:1 followed by the ratio of 5:1:1 (Table 4). When Induced Sterility (IS) from each mating ratio was compared, it was found that IS was significantly increased when the mating ratio increased, starting from the ratio of 1:1:1 onwards (Table 4). When Fried Index (C) from each mating ratio was compared, it was found that C significantly increased when the mating ratio increased at the ratio of 1:1:1 to 5:1:1, then it dropped again at the ratio of 10:1:1 and it increased at the ratio of 20:1:1 (Table 4).

In conclusion, ♂ ir-w showed the highest male competitiveness (C = 0.71) when compared to those of ♂ w (C = 0.35) and ♂ ir at the ratio of 1:1:1 (C = 0.17), but still they were all less competitive than those of the wild males. However, when the ratios were increased starting from 10:1:1 onwards, ♂ w showed the highest competitiveness and they were almost two times more competitive than those of wild males when the former were released at the ratio of 20:1:1.

### Comparison of survival rate and longevity between irradiated (SIT) and *Wolbachia* trans-infected (IIT) *Aedes aegypti* mosquitoes

In these experiments, 30 male and 30 female mosquitoes were separately put inside a mosquito 30 cm x 30 cm x 30 cm cage and 10% of sucrose solution was provide inside the cage. The number of dead mosquitoes were recorded and removed from the cage daily. The experiment was terminated when mosquitoes in the cage were all dead. Survival rate and longevity of ♂nr vs ♀ nr; ♂ir vs ♀ir, ♂nw vs ♀ nw, and ♂w vs ♀w were compared.

**Fig 4:**
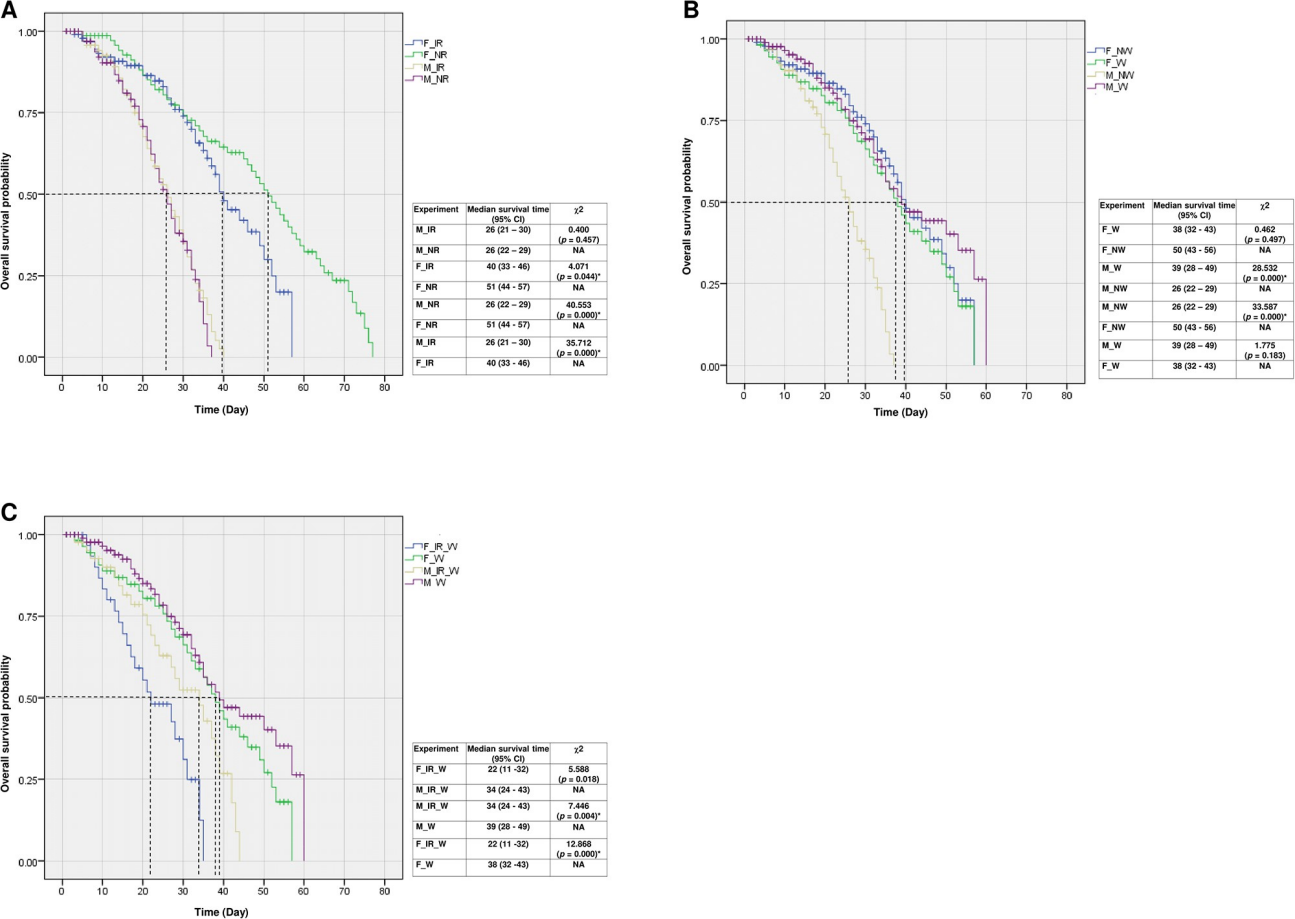
Kaplan Meir survival of non-irradiated (nr) and irradiated (r) (A), *Wolbachia*-uninfected (nw) and *Wolbachia* trans-infected (w) (B), and non-irradiated *Wolbachia* trans-infected (nr-w) and irradiated *Wolbachia* trans-infected (ir-w) *Aedes aegypti* male and female mosquitoes after being exposed to radiation at 50 Gy.

Results showed that when comparing the survival rate between ♂ nr vs ♂ ir, the survival rate was nearly the same and no significant difference was observed (*p* > 0.05) (Fig 4A). For females, it was found that the survival rate of ♀ ir was significantly reduced when compared to those of the control (*p* < 0.05) (Fig 4A).

When non-irradiated mosquitoes were considered, it was found that ♂ nr showed a significant lower survival rate when compared to those of ♀ nr (*p* < 0.05) (Fig 4A). When irradiated mosquitoes were considered, it was found that the survival rate of ♀ ir was significantly increased when compared to those of ♂ ir (*p* < 0.05) (Fig 4A). In conclusion, irradiation had no impact on both the survival of irradiated males, since irradiated males showed no significant difference in survival rate when compared to those of the non-irradiated males. Contrary to the males, irradiation significantly reduced the survival rate of irradiated females when compared to those of non-irradiated females.

The same experiments were done with *Wolbachia*-uninfected and *Wolbachia* trans-infected mosquitoes. The results showed that the survival rate of ♂ w was significantly increased when compared to those of the control (♂ nw) (*p* < 0.05) (Fig 4B). For the females, it was found that the survival rate of ♀ w was not different from those of ♀ nw (*p* > 0.05). When *Wolbachia*-uninfected mosquitoes were studied, it was found that survival rate of ♂ nw was significantly decreased when compared to the those of ♀ nw (*p* < 0.05) (Fig 4B). When *Wolbachia* trans-infected mosquitoes were studied, it was found that survival rate of ♂ w was almost the same when compared to those of ♀ w (*p* > 0.05). In conclusion, our results showed that *Wolbachia* infection significantly increased survival rate of *Wolbachia* trans-infected males but it reduced survival rate of *Wolbachia* trans-infected females.

When irradiation was combined with *Wolbachia* trans-infection, it was found that the survival rate of ♀ ir-w was significantly reduced when compared to those of ♂ ir-w (*p* < 0.05) (Fig 4C). When only the males were considered, it was found that the survival rate of ♂ ir-w was significantly reduced when compared to those of ♂ w (*p* < 0.05) (Fig 4C). For females, the survival rate of ♀ ir-w was also significantly reduced when compared to those of ♀ w (*p* < 0.05) (Fig 4C). In conclusion, irradiation when combined with *Wolbachia* trans-infection, it significantly reduced the survival rate of both *Ae*. *aegypti* males and females, and this reduction was more enhanced than the effect of *Wolbachia* trans-infection alone.

## Discussion

In the current study, we found that irradiated *Wolbachia* trans-infected *Ae*. *aegypti* males were twice as competitive as wild males (C = 0.71) when compared to *Wolbachia* trans-infected males (C = 0.35), where the latter was twice as competitive when compared to those of irradiated males (C = 0.17) at the ratio of 1:1 sterile to wild males. A competitiveness index of at least 0.2 was considered acceptable with a ratio between sterile to fertile males of 1:1 in Tephritid flies SIT programs [41]. Moreover, the C value of 0.2 was the lower threshold for cost-effective projects, as the lower value would require an asymptomatic increase in the amount of sterile males to be released [42]. Our results were in agreement with the study of Atyme who showed that incompatible male mosquitoes were slightly superior to irradiated males for mating competitiveness [28]. As the irradiated *Wolbachia* trans-infected males were the highest competitive in this study, this could be the benefit of combined IIT/SIT approach.

Our finding including many studies revealed a reduction of male competitiveness in *Wolbachia*-infected male mosquitoes [43, 44]. However, our study contradicted the study of Segoli [45] who reported that *Wolbachia*-infected males were equally successful to uninfected males in mating. *Wolbachia* was found in various parts of their hosts [46] and their effects on behavior of the hosts were varied, therefore, the role of *Wolbachia* in mediating mating behavior of their hosts remains controversial [47]. In terms of irradiation, our study was in agreement with many studies showing a reduction or negative relationship between male competitiveness and irradiation in mosquitoes [48–50], but it contradicted some studies that showed no effect of irradiation on the mating of mosquitoes [51]. Irradiation induced chromosomal damage in the germ cells [52, 53] leading to desired sterility [52], but it also caused deleterious somatic effects that reduced the competitiveness of the males. Increase the number of sterile males released could compensate for reduced competitiveness but it does not make them more competitive. When sterile males were released in the environment, we suggested the released number between 5–10 times higher than the numbers of wild males for *Wolbachia* trans-infected males; 10–20 times for irradiated males, and 20 times for irradiated *Wolbachia* trans-infected males in order to increase the chance for wild females to encounter and copulate with sterile males rather than with the wild males.

In this study, we found that complete male sterility (no hatched eggs) was induced by either irradiation or *Wolbachia* trans-infection; but when irradiation and *Wolbachia* trans-infection were combined, high level of sterility was observed but still some eggs were hatched, although with a very low egg hatch rate. Irradiation-induced dominant lethal mutations in germ cells were the main cause of male sterilization in the SIT approach, however, cell damage could be manifested by a decrease in performance traits in the irradiated mosquitoes [10]. For IIT approach, male sterility was induced from sperm-egg incompatibility occurring when *Wolbachia*-infected males mated with uninfected females or females infected with an incompatible *Wolbachia* strain [21]. From this perspective, we could assume that sterility induced by IIT approach seemed to cause less damage to insects when compared to those of SIT. However, when female sterility was taking into consideration, IIT approach alone could not be enough to induce complete female sterility because *Wolbachia* trans-infected females could still lay eggs and those eggs could still hatch with high egg hatch rate. This could pose risk of population replacement in case if the *Wolbachia* trans-infected females were accidentally released in the environment. Continued releases of *Wolbachia* infected females led to population replacement [22, 54]. Risk of population replacement caused by an unintentional release of *Wolbachia*-infected females during male releases for population suppression has been a concern and an obstacle in implementing the IIT method in nature, therefore, combining IIT/SIT could be a safer method to minimize the consequences of inadvertent female release [26].

The combination of IIT and SIT strategy could be applied to any targeted species for which an adequate sexing system is not available [55, 56]. However, the optimal radiation dose chosen for insects that are to be released should ensure a balance between the radiation dose, the induced sterility, the competitiveness of the male, and the final level of reproductive sterility induced in the wild females [57]. In this study, we found that irradiation dose of 50 Gy could significantly induced complete sterility in irradiated males. The same irradiation dose also induced complete sterility in irradiated *Wolbachia* trans-infected females, hence risk of population replacement due to an unintentional release of *Wolbachia*-infected females was diminished or almost negligible for combined IIT/SIT approach. The irradiation dose which obtained 99% male sterility while maintained male mating competitiveness or without significantly reduction in their biological quality was suggested [42, 58]. Besides, we found that the same irradiation dose showed no effect on flight ability nor the survival rate of irradiated males. The latter finding was in agreement with many studies [59, 60]. Moreover, we found that irradiated *Wolbachia* trans-infected males showed slightly shorter survival rate (34 days) when compared to those of *Wolbachia* trans-infected males (39 days), but still the former showed largely longer survival rate when compared to those of irradiated males (26 days). In contrary, we found that irradiated *Wolbachia* trans-infected females showed the shortest survival rate (22 days) when compared to irradiated (40 days) and *Wolbachia* trans-infected (38 days) females. This finding exhibits advantages of the combined IIT/SIT as this approach not only reduced risk of population replacement, but somehow, it also shortened the survival rate of irradiated *Wolbachia* trans-infected females, therefore, it was considered as safe for implementation as an alternative vector control program.

## Supporting information

S1 Fig Dose response curve of irradiated *Aedes aegypti*. (PNG)

S1 Table Results of *Wolbachia* detection. (DOCX)

S2 Table Analysis of variance of *Wolbachia* density. (DOCX)

S1 Data Data used to generate S1 Table. (XLSX)

S2 Data Data used to generate S2 Table. (XLSX)

S3 Data Data used to generate Table 2. (XLSX)

S4 Data Data used to generate Table 3. (XLSX)

S5 Data Data used to generate Table 4. (XLSX)
