# Supplementary figures and images for: Comparison on the quality of sterile Aedes aegypti mosquitoes produced by either radiation-based sterile insect technique or Wolbachia-induced incompatible insect technique
Source: PLoS One. 2025 Feb 12;20(2):e0314683. doi: 10.1371/journal.pone.0314683 (PMC11819552; doi:10.1371/journal.pone.0314683)

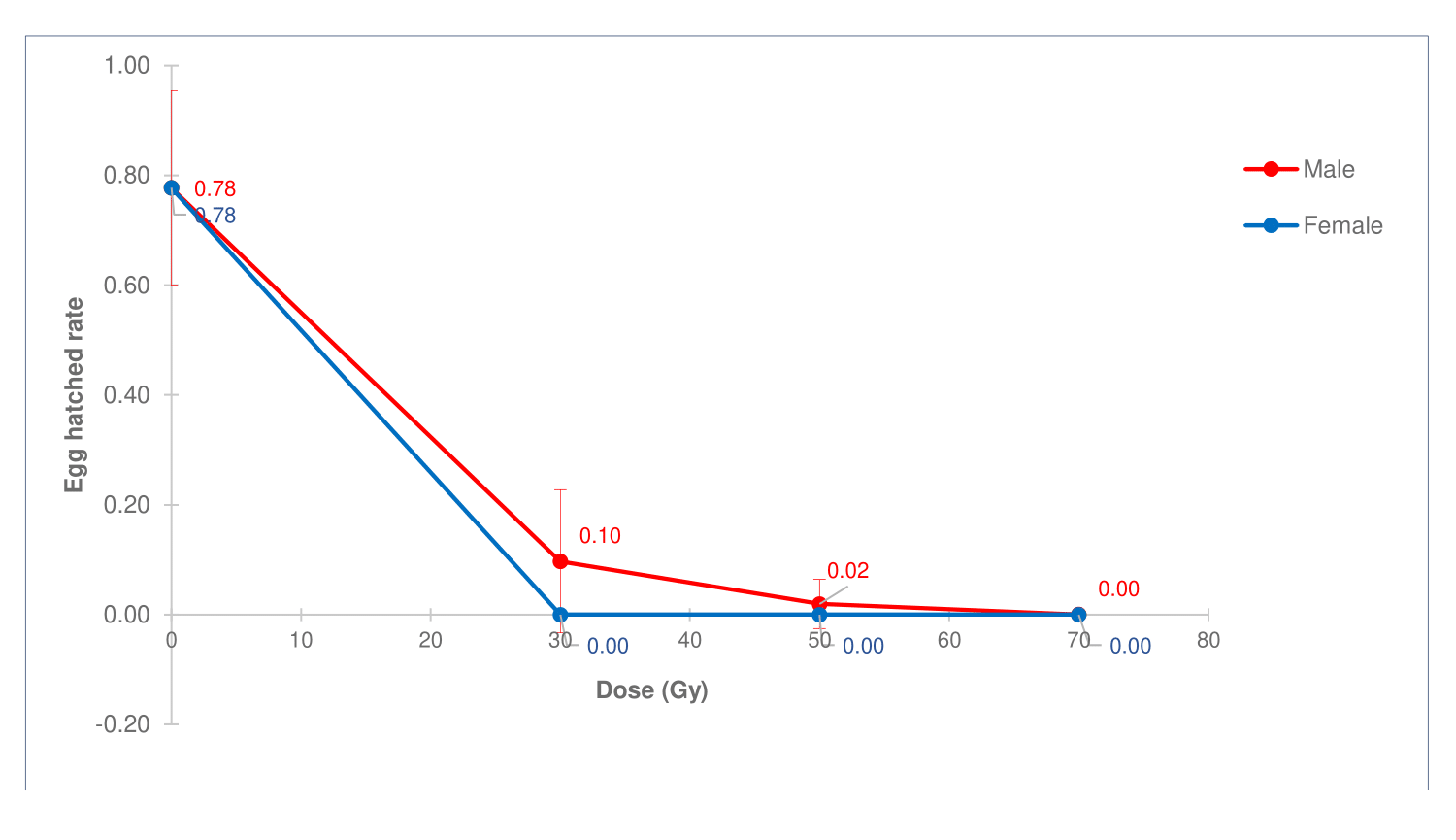

Supplement: S1 Fig — (PNG) [file pone.0314683.s001.png]
