# Supplementary material for: Comparison on the quality of sterile Aedes aegypti mosquitoes produced by either radiation-based sterile insect technique or Wolbachia-induced incompatible insect technique
Source: PLoS One. 2025 Feb 12;20(2):e0314683. doi: 10.1371/journal.pone.0314683 (PMC11819552; doi:10.1371/journal.pone.0314683)
Supplement: S1 Table — (DOCX) [file pone.0314683.s002.docx]

**Detection of *Wolbachia* in *Wolbachia* trans-infected *Aedes aegypti***

In these experiments, a total of 196 male and female *Wolbachia* trans-infected *Ae. aegypti* mosquitoes from 4 production batches (F6-F9), averaging 49.00 ± 28.44 mosquitoes per lot, were sampled for *Wolbachia* detection using PCR. Results showed that the *w*AlbB *Wolbachia* strain had been detected in all mosquito samples in every production lot or in 100% of male and female *Wolbachia* trans-infected mosquitoes (S1 Table, Supplementary Data). Therefore, it could be concluded that the *w*AlB strain was stable in the *Wolbachia* trans-infected *Ae. aegypti* up to at least 9 generations. However, *Wolbachia* density needed to be further investigated in order to assess the quality control of *Wolbachia* trans-infected *Ae. aegypti* mosquitoes.

**S1 Table. Results of *Wolbachia* (*w*AlbB) detection in *Wolbachia* trans-infected *Aedes aegypti* mosquitoes using PCR.**

| **Lot no.** | **N** | **No.**  **Male** | **No.**  **female** | ***w*AlbB*-*positive**  **(%)** |
| --- | --- | --- | --- | --- |
| 1 | 45 | 23 | 22 | 100.00% |
| 2 | 22 | 11 | 11 | 100.00% |
| 3 | 89 | 45 | 44 | 100.00% |
| 4 | 40 | 20 | 20 | 100.00% |
| **Mean** | **49.00±28.44** | **24.75±14.43** | **24.25±14.01** | **100.00±0.00%** |
