# Supplementary material for: Comparison on the quality of sterile Aedes aegypti mosquitoes produced by either radiation-based sterile insect technique or Wolbachia-induced incompatible insect technique
Source: PLoS One. 2025 Feb 12;20(2):e0314683. doi: 10.1371/journal.pone.0314683 (PMC11819552; doi:10.1371/journal.pone.0314683)
Supplement: S2 Table — (DOCX) [file pone.0314683.s003.docx]

**S2 Table. Analysis of variance of *Wolbachia* density between irradiated *Wolbachia* trans-infected *Aedes aegypti* (♂ ir-w) males and (♀ ir-w) females when compared to those of control (♂ nr-w) males and (♀ nr-w) females.**

| **Experiment** | **N** | **Mean** ± **SD** | **95% CI** | **T** | **df** | ***P*** |
| --- | --- | --- | --- | --- | --- | --- |
| ♂ nr-w | 6 | 99.28 ± 21.90 | 32.68 – 78.29 | 6.255 | 5 | 0.002* |
| ♂ ir-w | 6 | 43.80 ± 1.82 |  |  |  |  |
| ♀ nr-w | 6 | 143.00 ± 128.03 | -113.99 – 210.87 | 0.767 | 5 | 0.478 |
| ♀ ir-w | 6 | 94.56 ± 46.35 |  |  |  |  |

* Significant difference at *p* < 0.05
